# Antimicrobial resistance in healthcare, agriculture and the environment: the biochemistry behind the headlines

**DOI:** 10.1042/EBC20160053

**Published:** 2017-03-03

**Authors:** Henrietta Venter, Michael L. Henningsen, Stephanie L. Begg

**Affiliations:** School of Pharmacy and Medical Sciences, Sansom Institute for Health Research, University of South Australia, Adelaide, SA 5000, Australia

**Keywords:** antimicrobial resistance, antiseptic, cross-resistance, hospital acquired infections, last resort antibiotic, spread of resistance

## Abstract

The crisis of antimicrobial resistance (AMR) is one of the most serious issues facing us today. The scale of the problem is illustrated by the recent commitment of Heads of State at the UN to coordinate efforts to curb the spread of AMR infections. In this review, we explore the biochemistry behind the headlines of a few stories that were recently published in the public media. We focus on examples from three different issues related to AMR: (i) hospital-acquired infections, (ii) the spread of resistance through animals and/or the environment and (iii) the role of antimicrobial soaps and other products containing disinfectants in the dissemination of AMR. Although these stories stem from three very different settings, the underlying message in all of them is the same: there is a direct relationship between the use of antimicrobials and the development of resistance. In addition, one type of antimicrobial could select for cross-resistance to another type and/or for multidrug resistance. Therefore, we argue the case for increased stewardship to not only cover clinical use of antibiotics, but also the use of antimicrobials in agriculture and stewardship of our crucially important biocides such as chlorhexidine.

## Introduction

The serendipitous discovery of penicillin by Alexander Fleming, and subsequent development of the fungal metabolite into a viable treatment for infections by Howard Florey and his group, marked the beginning of the golden age of antibiotics. Huge optimism reigned during the 1960s and into the 1970s due to the defeat of smallpox and polio. However, less than 50 years later the WHO published its first Global Report on Antimicrobial Resistance and concluded that, without intervention, we are heading for a post-antibiotic era, where minor infections and small injuries will once again be fatal [1]. Later that year the first economic report on the impact of antimicrobial resistance (AMR), commissioned by David Cameron (the then Prime Minister of Britain) and headed by economist Lord O’Neill, was published [2]. According to the O’Neill report, if nothing is done, antibiotic resistance-related deaths would increase from 700000 annually to 10 million annually by 2050, overtaking cancer as the main cause of mortality and costing the healthcare industry trillions of USD [2].

Microorganisms are incredibly adaptable, and considering that *Escherichia coli* can divide every 20 min under ideal conditions, this gives them a huge evolutionary advantage over humans with an average lifespan of 75 years. They can respond and resist any treatments we might challenge them with. Most of our antibiotics come from other microorganisms [3]. This is hardly surprising as they excrete these compounds to fight each other in their continuous competition for resources. This also provides an explanation for the discovery of resistance genes in 30000-year-old permafrost [4], making the development of resistance against antimicrobials unsurprising. What is astounding though, is the immense speed with which resistance is developing. There is no doubt that human intervention plays a substantial role in the development of AMR, as there is a linear correlation between the use of antibiotics and the development of resistance [5,6]. Moreover, if the use and abuse of antibiotics in healthcare settings is alarming, it is worth pausing to consider the fact that animals in the U.S.A. consume more than twice as many medically important antibiotics as humans [7]. This alarm is compounded by the fact that in many countries, several tonnes of last-resort antibiotics, such as colistin, are used in animal feed every year [8–10]. Although antibiotics are used therapeutically in food animals to treat clinical disease, they are also applied prophylactically to prevent common disease outbreaks (particularly in intensively farmed animals) and subtherapeutically to enhance animal growth [11,12]. As for the use of antimicrobials in general household items, there is no limitation (or even quantification) of the amounts used, and the ability of these antimicrobials to hasten the development of resistance has gone largely unnoticed.

In this review, we will look at some of the stories on AMR that have made headlines recently. An analysis of the development of the resistance will be provided and the biochemical mechanism underlying the observed resistance will be explored. The current state of our preventative measures to curb the threat of AMR will also be evaluated.

## Hospital-acquired infections

Hospital-acquired infections frequently make news headlines and gain considerable public interest. These infections are caused by a range of opportunistic pathogens (organisms that only cause disease in immunocompromised individuals); many of them multidrug resistant [13]. Device-associated infections such as ventilator-associated pneumonia (VAP) and urinary tract infections (UTI) account for approximately 60% of all hospital-associated infections [13,14]. A meta-analysis found that in the United States, the treatment of hospital-acquired infections cost an estimated $10 billion annually [15].

We will focus on the most notorious and widespread hospital-acquired infection [16], responsible for nearly 20000 in-hospital deaths every year in the U.S.A. alone [17], namely methicillin-resistant *Staphylococcus aureus* **(**MRSA), and explore the biochemistry behind the development of resistance in this pathogen.

### Methicillin-resistant *S. aureus*

**Figure 1:**
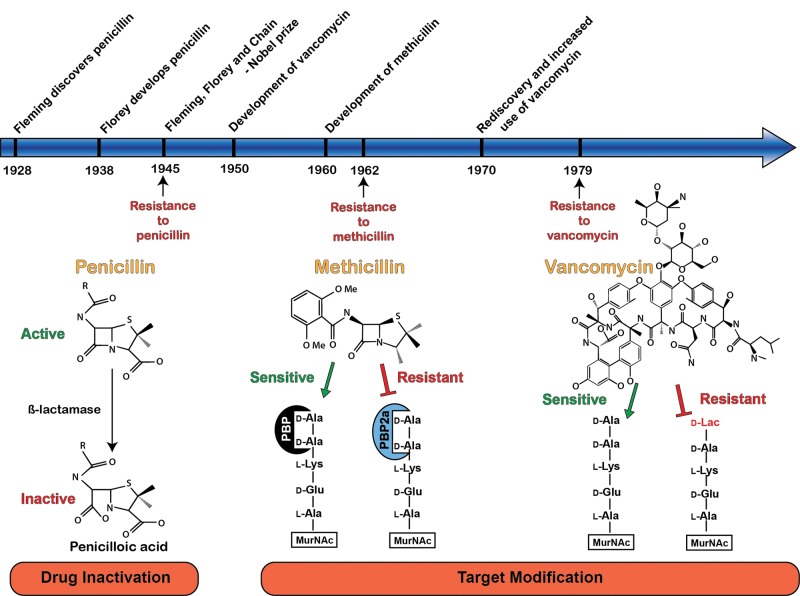
Timeline of selected antibiotic development and reported resistance A mechanism of resistance is illustrated for each antibiotic. Penicillin is commonly inactivated by bacterial β-lactamases, which cleave the β-lactam ring, forming the inactive penicilloic acid. Subsequent development of methicillin utilized a larger aryl side chain that was largely resistant to hydrolytic cleavage by β-lactamases. Instead, resistance to methicillin is driven by the expression of the alternative transpeptidase, PBP2a, which has a lower affinity for methicillin and can catalyse peptidoglycan cross-linking despite methicillin intervention. Resistance to vancomycin is driven by structural alteration of the terminal dipeptide that is modified from d-alanyl-d-alanine (d-Ala-d-Ala) to d-alanyl-d-lactate (d-Ala-d-Lac), reducing the affinity of the dipeptide for vancomycin and preventing disruption of peptidoglycan cross-linking.

*S. aureus* is a Gram-positive bacterium that causes skin and wound infections, bacteraemia and toxic shock syndrome [18–20]. Historically, *S. aureus* infections have been associated with severe morbidity and mortality, particularly in the wounds of soldiers, which were highly prevalent in the early 20th century [21]. During World War II, however, *S. aureus* wound infections were treated successfully for the first time with the newly developed antimicrobial, penicillin (Figure 1) [22]. Unfortunately, by the end of the war and in the same year that Alexander Fleming, Howard Florey and Ernst Chain received their Nobel Prize for the discovery and development of penicillin, the first strains of *S. aureus* resistant to penicillin started to emerge (Figure 1) [19,21]. Resistance to penicillin is through the acquisition of the *blaZ* gene, which encodes β-lactamase [23]. This gene product enzyme hydrolyses the generic β-lactam structure of penicillin, which is core to all β-lactam antibiotics, rendering them ineffective as a clinical treatment (Figure 1). The transmission of the *blaZ* gene is achievable through conjugal transfer, which resulted in widespread *S. aureus* penicillin resistance [23]. Resistance towards penicillin necessitated the development of methicillin, a narrow spectrum β-lactam alternative to penicillin. Methicillin and the penicillins that followed (oxacillin, nafcillin, cloxacillin and dicloxacillin) were designed with bulky side groups so that they would not fit in the active site of the β-lactamase and hence could not be inactivated by these enzymes (Figure 1) [24]. However, resistance towards methicillin was first documented just 2 years after its clinical introduction, giving rise to MRSA. Methicillin resistance occurs through the acquired alternative penicillin-binding protein, penicillin-binding protein 2a (PBP2a), encoded by the *mecA* gene that is regulated by the *blaZ-blaI-blaR1* and *mecA-mecI-mecRI* systems [23,25]. In methicillin-sensitive *S. aureus*, the antimicrobial activity of methicillin is mediated through high-affinity interaction with the bacterial PBPs. This binding event ultimately results in inhibition of the PBP enzymes, preventing cross-linking of bacterial peptidoglycan and leading to cell lysis [25]. However, the PBP2a variant has a much lower binding affinity for methicillin, allowing its continued activity even in the presence of methicillin and therefore, allowing bacterial survival [24]. MRSA strains are highly resistant against most β-lactams and many other classes of antibiotics. Vancomycin is the drug of choice for the treatment of infections caused by methicillin-resistant staphylococci [26,27]; however, *S. aureus* strains with increased MICs against vancomycin are also emerging now.

### Vancomycin intermediate *S. aureus* (VISA) and vancomycin-resistant *S. aureus* (VRSA)

Just like the β-lactam antibiotics, vancomycin also inhibits cell wall synthesis. However, it has a different target. Vancomycin binds to the D-Ala-D-Ala residues that are part of the building blocks of the peptidoglycan cell wall, and so prevent the formation of the essential peptide cross-links used to connect the units of the cell wall [28]. This mechanism of inhibition was thought to represent a major breakthrough in the field of antibiotics, as resistance towards vancomycin was absent for many years after its introduction. However, a strain of *S. aureus* with reduced vancomycin susceptibility (VISA) was first reported in 1997 [29]. Since then the incidence of VISA has been steadily increasing so that VISA is currently an immediate concern in the treatment of infections caused by *S. aureus* [30,31]. VISA strains have thicker cell walls compared with vancomycin-sensitive strains. These conditions restrict vancomycin to the outer layers of bacterium and prevent it from inducing its antimicrobial effects [32,33]. VRSA strains have also emerged [29]. MRSA obtains resistance to vancomycin through the conjugal transfer of the plasmid borne transposon Tn1546 from the vancomycin-resistant *Enterococcus faecalis*. The specific biochemical mechanism conferred by the Tn1546 transposon was shown to alter the dipeptide residue D-Ala-D-Ala to D-Ala-D-Lac, a dipeptide with substantially lower affinity for vancomycin (Figure 1) [34]. Only a few cases of VRSA have been reported so far and therefore it does not represent an urgent public health threat.

The worldwide concern about the prevalence of MRSA and VISA meant that considerable effort went into developing alternatives to vancomycin for the treatment of MRSA. Currently, the advanced generation cephalosporin ceftaroline, the lipopeptide daptomycin, the vancomycin analogues telavancin, oritavancin and dalbavancin, and the oxazolidinones linezolid and tedizolid, can still be used against MRSA [35–37]. However, the development of resistance has been observed against these drugs already [38–40]. Therefore, the significance of MRSA and VISA should not be underestimated, due to both its significance regarding global mortality and its historical ability to develop resistance mechanisms in the presence of antibiotic stress.

## Resistance through the use of antibiotics in veterinary science and agriculture

### Apocalypse pig and the demise of a last resort antibiotic

**Figure 2:**
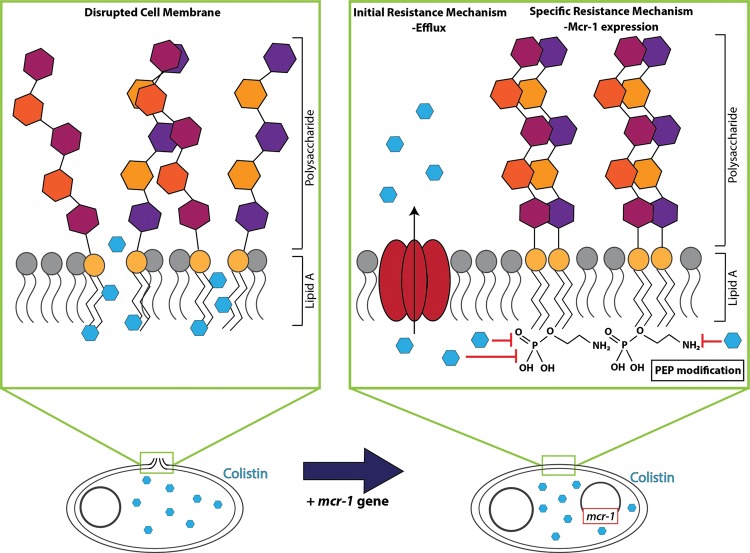
Selected mechanisms of colistin resistance The bactericidal activity of colistin relies on disruption of the bacterial cell membrane, initiated by electrostatic interaction between colistin and the lipid A portion of bacterial LPS. Immediate, albeit non-specific, resistance to colistin is mediated through transcriptional up-regulation of drug efflux pumps. Specific resistance to colistin is facilitated by the plasmid-mediated *mcr-1* gene, encoding a phosphoethanolamine transferase, which modifies lipid A with a phosphoethanolamine (PEP) group, preventing interaction between colistin and lipid A.

‘Apocalypse pig’ refers to the first pig reported to harbour a Gram-negative organism resistant to the last resort antibiotic, colistin [41]. The term ‘last resort antibiotic’ refers to an antibiotic that still has activity against resistant pathogens and are therefore used as a last line of treatment when other antibiotics fail. Once organisms have developed resistance against a last resort antibiotic, hardly any treatment options remain [42]. Colistin has an interesting history. It is a polymyxin type of antibiotic (also known as polymyxin E) that act by permeabilizing the outer membrane of Gram-negative organisms [43]. Electrostatic interactions between colistin and the lipid A subunits present in the lipopolysaccharide (LPS) of the outer membrane [43–45] alter the structural integrity of LPS, leading to cellular membrane permeability and resulting in bacterial death (Figure 2) [46,47]. Colistin is characterized by remarkable antimicrobial activity against hard to treat Gram-negative organisms such as multidrug-resistant *Pseudomonas aeruginosa, Acinetobacter baumannii* and *Klebsiella pneumoniae* [48–50]. It was first developed in the 1950s but was shelved in the 1970s due to its nephrotoxicity [45]. When widespread resistance developed against other antibiotics, Li et al. [51] revisited colistin and provided the pharmacokinetic and pharmacodynamic data that enabled the development of colistin for new clinical applications. Although colistin can be dosed as the parent compound, more commonly it is administered as a prodrug, colistin methanesulfonate, leading to some confusion in dosing regimes.

Since colistin was not used clinically for approximately 30 years, resistance against colistin was very rare. Early research indicated that colistin resistance in *E. coli* and *Salmonella* isolates could be conferred by a mutation in the two-component PhoP-PhoQ and/or PmrA-PmrB systems [46]. These mutations produce structural modifications in the lipid A subunit that reduce electrostatic interactions between the positively charged amino groups of colistin and negatively charged phosphate groups of the lipid A subunits, preventing disruption of the cell membrane [47]. However, recently, the plasmid-encoded *mcr-1* gene was discovered in colistin-resistant, commensal *E. coli* strains from food animals in China [10]. This non-chromosomal mechanism of colistin resistance raised fears for the rapid spread of resistance, similar to the dissemination of the β-lactamase coding antibiotic resistance genes *bla*_KPC_ and *bla*_NDM-1_ [52–54]. In addition, colistin is also subject to efflux by drug efflux pumps that could give initial resistance to this antibiotic (Figure 2) [55–57]. Even though colistin was not used clinically for a long time, many countries have been using colistin in agriculture [9]. China currently represents the world’s largest user of colistin in livestock, dedicating 11942 tonnes to livestock agricultural feeds annually [10]. Colistin-resistant organisms have been isolated from people with no prior exposure to colistin, indicating the possibility for the *mcr-1* gene to spread from animals to humans. The detection of the *mcr-1* gene in multiple countries makes it difficult to pinpoint the origin of AMR. Retrospective analysis indicated that the *mcr-1* gene has been present since the 1980s in *E. coli* in China without causing serious issues [58]. However, it is clear that a global reassessment of the agricultural use of this crucially important, last resort antibiotic, is now imperative. To this extent, the use of colistin as a feed additive for animals has been banned in China effective from 1 November 2016 [12].

### Resistance from farm to fork

Several other outbreaks of infectious disease caused by multidrug-resistant organisms, acquired through food sources, have brought the issue of the use of antibiotics in agriculture firmly into public attention. In 2014, a multistate outbreak of multidrug-resistant *Salmonella heidelberg* in the U.S.A. was linked to consumption of chicken meat from one supplier [59]. Prophylactic use of antimicrobials in factory farmed chickens is huge, currently estimated at a global annual consumption of 148 mg of antibiotic per kg of animal produced [60], to prevent outbreaks in the crowded and unhygienic conditions. Incidences like these have prompted many public calls for ‘antibiotic-free meat’. However, it is not residual antibiotics in the meat that poses a problem, the real issue is the selection of multidrug-resistant superbugs in animals raised on antimicrobials. These animals could act as reservoirs of resistant organisms that could eventually find their way to human consumers, either through the environment or through direct contact [61–63].

There is a widespread belief that antimicrobials that are not currently in clinical use are fine to use as growth promoters in feed animals, as resistance to these compounds would not lead to resistance to clinically used antimicrobials. However, this argument does not hold true, as pathogens can express drug efflux pumps that can expel many different classes of compounds, including the antimicrobials used as feed additives [64–66]. Organisms that express these efflux pumps will subsequently be resistant against a multitude of antimicrobial compounds, including those used in healthcare. This efflux-mediated resistance would confer a fitness advantage to organisms, sufficient to allow survival in the presence of clinical antibiotics until specific resistance mechanisms have been acquired [67,68]. Animals that are ill should undoubtedly be treated with antimicrobials, even medically important ones. However, the use of antibiotics and other antimicrobials both prophylactically and as growth-enhancers needs to be re-considered.

These facts are slowly being recognized and acted upon. In 2015, the WHO released their ‘Global action plan’ on AMR. One of the objectives in this report is to ‘optimize the use of antimicrobial medicines in human and animal health’ (Objective 4) by, among other things, curbing the ‘inappropriate or unregulated use of antimicrobial agents in agriculture’ [69]. In June, 2015, the Australian government adopted a ‘One Health’ approach, where one of their objectives is ‘Surveillance of antimicrobial resistance and antimicrobial usage in human health and animal care’ [70]. These measures are timely and much needed. It would be ideal though if monitoring programmes were extended not only to the use of antibiotics, but also to include biocides as addressed in the following section.

## The issue with antimicrobial soaps

### The FDA (U.S.A.) ban the inclusion of triclosan in antibacterial soaps

**Figure 3:**
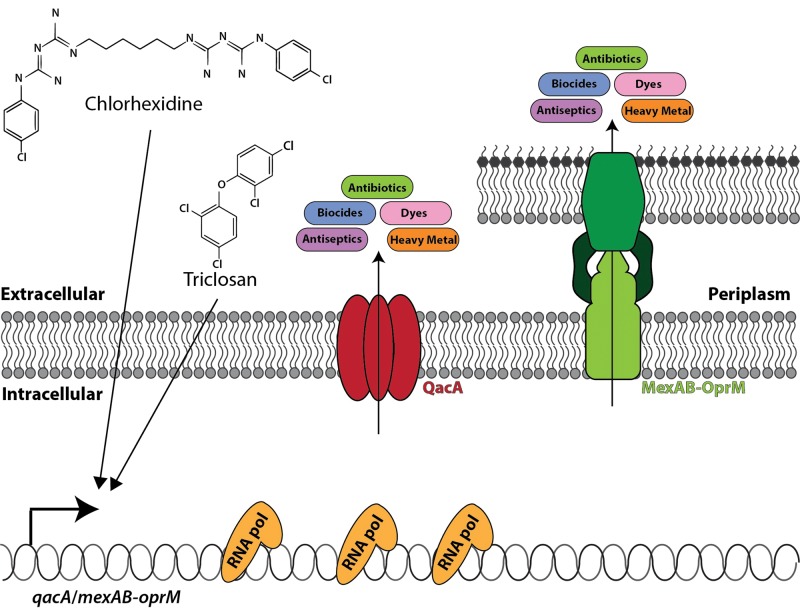
Biocide usage and antibiotic resistance Biocides such as triclosan and chlorhexidine exert their antimicrobial activity through non-specific interactions with cellular targets. An innate bacterial defence to toxic compounds, such as these, is up-regulation of multidrug efflux pumps, such as *qacA* in the Gram-positive organism *S. aureus* and *mexAB-oprM* in the Gram-negative organism *P. aeruginosa*. Once expressed, these efflux pathways will not only export biocides, but also antibiotics, antiseptics, heavy metals and dyes – hence resulting in the development of multidrug resistance.

Our society is obsessed with a sense of cleanliness and (understandably) infection control, as is evident by the widespread use of antibacterial soaps and the inclusion of antiseptics/biocides and antimicrobial nanosilver in many household cleaning and personal care products; from soaps to socks that prevent smelly feet. In September 2016, the FDA in the U.S.A. banned the use of the antibacterial agent triclosan as well as 18 other compounds from soaps [71]. There were several reasons for this ban. Firstly, triclosan containing soaps are not more efficient in preventing the spread of infection compared with normal soap [72]. Secondly, there are concerns regarding the safety and health effect of long-term exposure to triclosan [73]. Thirdly and very importantly, the inclusion of triclosan in soap could lead to the development of AMR [74]. Unlike antibiotics, biocides, antiseptics and nanosilver particles do not have specific targets, but act rather non-selectively on microbial cells. For this reason, it was believed that resistance should not easily develop against these types of compounds [75]. However, bacteria have a natural defence mechanism to protect them from toxic compounds: drug efflux pumps. These efflux pumps are membrane proteins that expel antibiotics and other toxic compounds from the microbial cells, thereby lowering their concentration inside the cell to sub-toxic levels (Figure 3) [68,72,73]. Drug efflux pumps are the main contributors to multidrug resistance by virtue of their ability to expel a wide variety of structurally and functionally distinct antibiotics. The substrate range of drug efflux proteins is not limited to antibiotics, but include biocides, dyes, detergents, heavy metals and endogenous compounds such as virulence factors [76–82]. Therefore, efflux pumps have the ability to give widespread resistance against these compounds, and simultaneously render the organisms resistant against the antibiotics that are currently in clinical use to treat infections [83–92].

### Use of chlorhexidine selects for resistance against the last resort antibiotic colistin

In contrast with triclosan, the biocide chlorhexidine is highly efficient in the prevention and control of the spread of infectious organisms [93]. It is widely used in various clinical applications that require decolonization and infection control. For example, chlorhexidine gluconate (CHG) is used as an antiseptic to decolonize skin before operations [94], as a mouthwash to prevent plaque formation and sterilization of the mouth before dental implants [95], as a daily bath solution to prevent hospital-acquired infections in e.g. burn patients [96] or as a rinse to prevent infections associated with indwelling devices such as VAP or UTI [94]. Resistance to CHG will therefore have dire consequences for infection control in hospital settings. Although CHG is mostly still very effective, and resistance to the high concentrations (1–2%) used in hospital disinfectants has not been reported for Gram-positive organisms, increased tolerance (in some cases up to 100-fold increase in MIC to CHG) has been reported in Gram-negative organisms, such as *P. aeruginosa* and *K. pneumoniae* [97–99]. More worryingly, a pan drug resistant strain of *K. pneumoniae* has been identified that was able to multiply in a 1% CHG disinfectant solution [100]. Resistance to CHG usually arise through the expression of drug efflux proteins that can pump out CHG such as QacA/QacB from *S. aureus* and the MexAB-OprM system from *P. aeruginosa* [88]. This also poses the question as to the possible relationship between CHG tolerance and antibiotic resistance. Very recently, links between the use of CHG and resistance to both vancomycin and the last line antibiotic, colistin, have been established [97,101]. These worrying observations clearly indicate that in addition to antibiotic stewardship, we also need stewardship of our biocides especially the critically useful ones such as CHG.

## Conclusions

We grew up in the golden age of antibiotics. A world without effective antimicrobials – where a simple scratch could cost you your life and where most modern medical procedures would no longer be possible – is unthinkable. Yet, we are bombarded on a daily basis with reports of AMR superbugs impervious to our best treatments. In this review, we explored the biochemistry behind reports on AMR in healthcare, agriculture and the environment. The underlying, unifying, factor of all these case studies is the fast development and global spread of AMR regardless of the geographical location or community of origin. This is a truly global problem that will only be solved by a global response.

The recent commitment by Heads of State at the UN general assembly to adopt a broad, coordinated approach to tackle AMR (http://www.un.org/pga/71/2016/09/21/press-release-hl-meeting-on-antimicrobial-resistance/) is therefore a much needed and a very timely global incentive. Good stewardship is needed not only in the medical use of antimicrobials, but also for the use of antimicrobials in animal health, the abundant use of antimicrobials in agriculture, and the widespread use of biocides and antiseptics in common household products. Only once these issues have been suitably addressed can we hope to slow the ever-increasing development of AMR.

“There is probably no chemotherapeutic drug to which in suitable circumstances the bacteria cannot react by in some way acquiring ‘fastness’ [resistance].”

—Alexander Fleming, 1946

## Summary

- Hospital-acquired infections remain a serious threat as increased resistance reduces or eliminates treatment options and costs billions of dollars per year to manage.
- The use of antibiotics in agriculture results in the development of multidrug-resistant organisms that act as reservoirs of resistance. These organisms or their genes can spread to humans either through direct contact or through the environment.
- The excessive use of antiseptics and biocides leads to resistance against these compounds and cross-resistance to antibiotics.
- Good stewardship programmes are needed, not only for clinically used antibiotics, but also for antimicrobials used in agriculture and for critically important antiseptics such as chlorhexidine.

## Abbreviations

AMR: antimicrobial resistance
CHG: chlorhexidine gluconate
LPS: lipopolysaccharide
MIC: Minimum inhibitory concentration
MRSA: methicillin-resistant *Staphylococcus aureus*
PBP: penicillin-binding protein
UTI: urinary tract infections
VAP: ventilator-associated pneumonia
VISA: vancomycin intermediate *Staphylococcus aureus*
VRSA: vancomycin-resistant *Staphylococcus aureus*
